# Exploration of Elite Stilbene Synthase Alleles for Resveratrol Concentration in Wild Chinese *Vitis* spp. and *Vitis* Cultivars

**DOI:** 10.3389/fpls.2017.00487

**Published:** 2017-04-07

**Authors:** Xianbo Zheng, Jiangli Shi, Yinmei Yu, Yanlong Shen, Bin Tan, Xia Ye, Jidong Li, Jiancan Feng

**Affiliations:** ^1^College of Horticulture, Henan Agricultural UniversityZhengzhou, China; ^2^Henan Key Laboratory of Fruit and Cucurbit BiologyZhengzhou, China

**Keywords:** resveratrol, stilbene synthase, elite allele, grape, association analysis, SSR

## Abstract

Resveratrol contributes to a plant’s tolerance of various abiotic and biotic stresses and is highly beneficial to human health. A search for elite alleles affecting resveratrol production was undertaken to find useful grapevine germplasm resources. Resveratrol levels in both berry skins and leaves were determined in 95 grapevine accessions (including 50 wild Chinese grapevine accessions and 45 cultivars) during two consecutive years. Resveratrol contents were higher in berry skins than in leaves and in wild Chinese grapevines than in grapevine cultivars. Using genotyping data, 79 simple sequence repeat (SSR) markers linked to 44 stilbene synthase (*STS*) genes were detected in the 95 accessions, identifying 40 SSR markers with higher polymorphisms. Eight SSR marker loci, encompassing 19 alleles, were significantly associated with resveratrol content on (*P* < 0.001), and 5 SSR loci showed repeated associations. Locus Sh5 had four associations: three positive for allele 232 (including leaves in the 2 years) and one negative for allele 236 in four environments. Loci Sh9 and Sh56 for a total of 7 alleles exhibited positive effects in berry skins in the 2 years. In berry skins, locus Sh56 with positive effects was closely linked to *VvSTS27*, and locus Sh77 with negative effects to *VvSTS17*, importantly, the two candidate genes both were located on Chromosome 16. The SSR marker loci and candidate genes identified in this study will provide a useful basis for future molecular breeding for increased production of natural resveratrol and its derivatives.

## Introduction

Resveratrol (*trans*-3, 5, 4′-trihydroxystilbene) is a natural phytoalexin occuring in a limited number of plant species, including *Vitis* spp. (Langcake and Pryce, 1976). Stilbenes in grapevine are very complex, and 18 stilbene derivatives were also identified in two grape samples, including resveratrol and piceid (Flamini et al., 2013). Resveratrol and piceid, in both *cis* and *trans* have been characterized in wine and grape berry (Pezet et al., 1994; Lamuela-Raventos et al., 1995; Romero-Pérez et al., 2001; Vitrac et al., 2005). These compounds are formed by oligomerization of *trans*-resveratrol in grape tissues under stress conditions such as exogenous attack or pathogen infections (Cichewicz et al., 2000; Romero-Pérez et al., 2001). It is interesting to note that *trans*-resveratrol showed either lower or higher concentration in wine and berry using different determination methods, compared with *trans*-piceid (Lamuela-Raventos et al., 1995; Ribeiro de Lima et al., 1999; Romero-Pérez et al., 2001; Vian et al., 2005; Vitrac et al., 2005; Flamini et al., 2013).

Table grapes and wines are the main food sources of resveratrol. The studies have focused on *trans*-resveratrol due to its various physiological functions in consumers, including antioxidative, anti-tumor, anti-inflammatory activities and reduction of cardiovascular disease and obesity (Jang et al., 1997; Alonso et al., 2002; Frombaum et al., 2012; Konings et al., 2014). The accumulation of resveratrol in plant tissue is induced by exogenous hormone, pathogen attack and UV-C irradiation (Zheng et al., 2009; Shi et al., 2014; Wang et al., 2015, 2016; Yin et al., 2016).

Stilbene synthase (STS), a key enzyme in the biosynthesis pathway of resveratrol, belongs to the polyketide synthase family (Rupprich and Kindl, 1978). Experiments aimed at the generation of transgenic plants with increased resveratrol content or improved resistance to fungal pathogens have focused on inserting foreign *STS* genes, which were mostly from *Vitis vinifera* (Leckband and Lorz, 1998; Zhu et al., 2004; Serazetdinova et al., 2005; Cheng et al., 2016). Additionally, inserting a foreign *STS* gene also influenced piceid accumulation in transgenic lines (Ruhmann et al., 2006; Liu et al., 2011; Carlos-Hilario et al., 2015). Recent studies showed that the *STS* gene family from grapevine included 40 or so members (Parage et al., 2012; Vannozzi et al., 2012; Shi et al., 2014). A very recent report characterized the function of an *STS* allele (Jiao et al., 2016).

Although the identity and/or function of some members of the *STS* gene family have been demonstrated, little information is available on how allelic diversities among *STS* genes contribute to variation in resveratrol accumulation in *Vitis* germplasm. In our previous study, members of the *STS* gene family showed one of two expression patterns and different expression levels in response to powdery mildew (Shi et al., 2014). Examination of allelic variation and linkage disequilibrium by a candidate gene-based approach would help to decipher the genetic basis of resveratrol biosynthesis. To do this, a representative sample of 95 grapevine accessions were selected, comprising both wild Chinese and cultivated grapevines, both green- and red-skin berries, and both seedless and seeded berries. SSR markers (79 pairs) distributed over the known *STS* genes from the grapevine PN40024 genotype were designed. Association analysis between *STS* genes and resveratrol content was performed on this wide collection of wild Chinese grapevines and cultivated European grapevines in order to find the elite alleles responsible for resveratrol accumulation. The results identify grapevine resources that can be used to obtain new grapevine cultivars with high levels of resveratrol in their berries, and can provide useful information for further research on resveratrol biosynthesis.

## Materials and Methods

### Plant Materials and Treatments

Grape accessions, including 50 wild Chinese grapevine species and 45 cultivars from the European species *V. vinifera* or the American species *V. labrusca* (**Table 1**), were grown under natural field conditions at the National Grape Germplasm Resources Repository of Zhengzhou Fruit Research Institute, Chinese Academy of Agricultural Sciences. Warm temperate continental climate of Zhengzhou has clear four seasons. The average annual precipitation is about 630 mm and mean temperature is 14.4°C. The details of climatic data were shown in Supplementary Table S1. The experiment vines were planted 9 or 10 years ago in sandy fluvo-aquic soil. And no special cultural practices were taken. All of the vines were in good condition. Grape berries were collected from June to September and leaves were picked at the end of June in 2013 and 2014. Samples were harvested from three grape vines for each accession. For the berries, three grape clusters on each plant were picked, one from the top, middle, and bottom of the canopy, respectively. To ensure that all berries were harvested at their full ripeness, we checked the seeds in the berries every 2 days from June till September. When the seeds completely ripened, the size of berries was no longer increasing, and the red grapes were fully colored, the berries were sampled from that accession. For the leaves, the second or third leaves (depending on healthiness) from the bottom of three different branches with more than 10 leaves were picked in the end of June. Unhealthy berries (cracking, smaller and other underdeveloped fruits) were removed before the samples were quickly frozen in liquid nitrogen and held at -80°C until use.

**Table 1:** Fifty wild Chinese grapevine accessions and 45 cultivars were used in this study.

No.	Species	Accession or cultivar	No	Species	Accession or cultivar
1	*V. labrusca*	Champion	49	*V. adenoclada*	Shuangxi 01
2	*V. vinifera*	Zhengguo 6	50		Shuangxi 03
3	*′′*	Jan-87	51		Zhijiangshui
4	*′′*	Amilia	52	*V. davidii*	Huitong No.1
5	*′′*	Guifeimeigui	53	*′′*	Huitong No.2
6	*′′*	Irsay Oliver	54	*′′*	Wuhan
7	*′′*	Olimpia	55	*′′*	Dongxiangjiao
8	*′′*	Baijixin	56	*′′*	Hongjiangyanlong 05
9	*′′*	Bolgar	57	*′′*	Hongjiangtongmu 07
10	*′′*	Pink varieties Taipei	58	*′′*	Zhijiang 01
11	*′′*	Mathias Aromatic	59	*′′*	Hongjiang 04
12	*′′*	Fenghuang 51	60	*′′*	Hongjiang 08
13	*′′*	Guibao	61	*′′*	Hongjiang 09
14	*′′*	Red Globe	62	*′′*	Hongjiang 10
15	*′′*	Malaga Rose	63	*′′*	Fuan
16	*′′*	Huangmisi	64	*′′*	Tangwei seedling
17	*′′*	Jingxiu	65	*′′*	Zhejiangtianmushan No.2
18	*′′*	Muscat Hamburg	66	*′′*	Zhejiangtianmushan No.3
19	*′′*	Manai	67	*′′*	Xiangzhenzhuhongye
20	*′′*	Munage	68	*′′*	Xiangzhenzhulvye
21	*′′*	Senio de Malingre	69	*′′*	Hunan
22	*′′*	Miskat Plevenski	70	*′′*	Gaoshan No.1
23	*′′*	Queen of Vineyard	71	*′′*	Gaoshan No.2
24	*′′*	Zhengguo 5	72	*V. amurensis*	S48-3
25	*′′*	Xiangfei	73	*′′*	N43-3
26	*′′*	Shenyangmeigui	74	*′′*	Changbai No.9
27	*′′*	Ribier	75	*′′*	Shuangyou
28	*′′*	Yangputao	76	*V. ficifolia*	946
29	*′′*	Yalishanda	77	*′′*	943
30	*′′*	Muscat MathiaszJanosne	78	*′′*	Qinling No.2
31	*′′*	Xiabai	79	*′′*	Wugang
32	*′′*	Italia	80	*′′*	Xinyang 01
33	*′′*	Zaomanao	81	*′′*	Fengjugou 02
34	*′′*	Zaotianmeiguixiang	82	*′′*	Fengjugou 03
35	*′′*	Zhengzhouzaoyu	83	*′′*	Shibanyan 02
36	*V. vinifera* x *V. labrusca*	Zifeng	84	*′′*	Shibanyan 05
37	*V. vinifera*	Zexiang	85	*′′*	Shibanyan 06
38	*′′*	Zijixin	86	*′′*	Shibanyan 08
39	*′′*	Jingzaojing	87	*′′*	Luoning 06
40	*′′*	Thompson Seedless	88	*′′*	Qinling 03
41	*′′*	Pinot Noir	89	*′′*	Jiuligou
42	*′′*	Cabernet Sauvignon	90	*V. betulifolia*	Songxian
43	*V. vinifera* x *V. amurensis*	Beimei	91	*V. romanetii*	Lingbao
44	*′′*	Beichun	92	*V. pseudoreticulata*	Huadong
45	*V. vinifera*	Zhengguodawuhe	93	*′′*	1057
46	*V. quinquangularis*	Guizhou	94	*V. yeshanensis*	Yanshan
47	*V. amurensis*	Baitianman 03	95	*V. adstricta*	Yingyu
48	*V. wilsonae*	Baotianman			

*Numbers 1–45 were *Vitis* cultivars, and Numbers 46–95 were wild grapevine species*.

### Determination of *Trans*-resveratrol Content by HPLC Method

*Trans*-resveratrol levels in berry skins and leaves were measured using HPLC as described by Li et al. (2006) with some modifications, in 95 grapevine accessions in 2013 and 2014. The standard for *trans*-resveratrol was purchased from Sigma–Aldrich (USA). Fruits were peeled and juice was soaked up using filter paper.

Three gram samples were ground to powder using a porcelain mortar and pestle in liquid nitrogen, extracted by 15 mL ethyl acetate in the dark at 25°C for 48 h, and centrifuged at 10,000 r⋅min^-1^ for 10 min. The supernatants were transferred into a tube containing 5 mL ethyl acetate, followed by centrifugation at 10,000 r⋅min^-1^ for 10 min. All supernatants were evaporated to dryness by Nitrogen blowing instrument (DCY-12S, Qingdao Haike, China) at 40°C. Dried samples were then dissolved in 2 mL of methanol and stored at -80°C. The samples were filtered through a 0.22 μm PTFE membrane filter before resveratrol analysis. Extractable amounts of resveratrol were analyzed using a Waters e2695 HPLC system (USA). Elution was carried out with a mobile phase delivered using a Waters C18 HPLC pump at a flow rate 0.8 mL⋅min^-1^. A Waters 2996 UV detector was used at 306 nm. Mean values and standard deviations were obtained from three biological replicates. An HPLC chromatogram of resveratrol was made with a standard solution. The resveratrol content was analyzed by Excel 2003 (Microsoft, USA) and SPSS 17.0 software (IBM, USA).

### DNA Isolation and PCR Amplification

Genomic DNA was extracted using Ezup Column Plant Genomic DNA Purification Kit following the manufacturer’s protocol (Sangon Biotech, Shanghai, China). The concentration of the extracted DNA was assessed using a Thermo ND 2000 spectrophotometer (ThermoFisher, USA). Genomic DNA was adjusted to a final concentration 50 ng/μL and was used for PCR amplification.PCR reactions were carried out in a final volume of 20 μL. Amplification reactions were carried out on a ABI Veriti thermal cycler (USA) using the following cycling profile: 95°C for 5 min, followed by 35 cycles at 95°C for 45 s, 48–56°C for 45 s, and 72°C for 1 min, and a final extension step at 72°C for 10 min. The amplification products were separated through polyacrylamide gel electrophoresis.

### Analysis of SSR Markers

Based on predicted *STS* gene sequences in the 12x grapevine PN40024 genome^1^ and the gene positions of these 44 *STS* genes (Shi et al., 2014), a total of 79 pairs of SSR primers on chromosomes 10 and 16 were designed using GRAMENE ssrtool^2^. Parameter settings were as follows: tetramer for the maximum motif-length group, and 4 for the minimum number of repeats.

Allelic variation was analyzed by calculating the number of alleles (Na), effective number of alleles (Ne), observed heterozygosity (Ho), and expected heterozygosity (He) using Popgene software. Polymorphism information content (PIC) was calculated using PIC-CALC.

Genetic distance matrices were obtained using SSR data in DPS software^3^. A phylogenetic tree was constructed by the unweighted pair-group method with arithmetic averages (UPGMA) with MEGA 6.0 software^4^.

### Population Structure and Association Analysis

Using 40 *STS*-gene-associated SSR markers, the genetic population structure of the 95 accessions was determined by Structure 2.1^5^. A burn-in phase of 10,000 iterations was followed by 100,000 Monte Carlo Markov Chain iterations. The optimal population number *k* (from 1 to 10 assumed in this study) was estimated (Evanno et al., 2005). Ten replicates were performed for each cluster, k. When an inflection emerged in the LnP (D) curve, the corresponding *k* value was adopted as the optimal group number. The values of the estimated membership probability (Q) were calculated to serve as covariates in the association analysis with general linear model (GLM) in Tassel 2.1^6^. Phenotypic effect values of some marker alleles were evaluated according to null allele as suggested by Breseghello and Mark (2006).

## Results

### *Trans*-resveratrol Content

**FIGURE 1:**
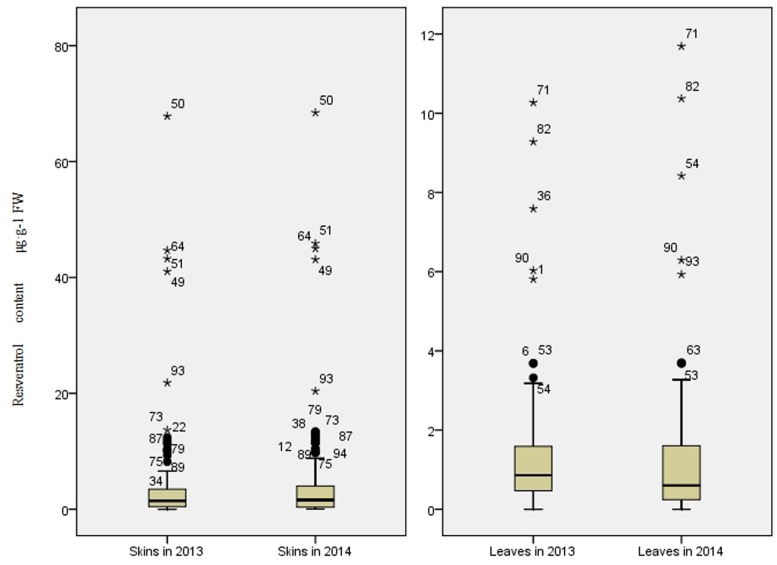
**Range and distribution of *trans*-resveratrol content in skins and leaves of 95 grapevine accessions (50 wild Chinese accessions and 45 grapevine cultivars) in 2013 and 2014**.

The *trans*-resveratrol levels in skin and in leaf collected from all accessions were determined by HPLC (**Figure 1**). The *trans*-resveratrol content in berry skins ranged from 0.05 to 67.82 μg⋅g^-1^ FW in 2013 and from 0.03 to 68.44 μg⋅g^-1^ FW in 2014. For both seasons, the highest levels were from the wild Chinese grapevine *V. adenoclada* accession Shuangxi 03. In leaves, the *trans*-resveratrol content ranged from 0.04 to 10.27 μg⋅g^-1^ FW in 2013 and from 0.09 to 11.69 μg⋅g^-1^ FW in 2014. The highest levels for both years were in leaves from wild Chinese grapevine *V. amurensis* accession Gaoshan No.2. Resveratrol contents were higher in berry skins than in leaves for each genotype.

Between the 2 years, the variation of resveratrol content was more stable in wild grapevine accessions than that of cultivated ones. More of the cultivated accessions (51%) showed year-to-year variations of resveratrol content in skin greater than 50%, compared to only 8% of wild grapevine ones, showing such large variations. Similarly, in leaves, 22% of wild accessions and 67% of cultivated ones showed resveratrol content variations greater than 50% (Supplementary Table S2). The results suggested that wild ones retained stable resveratrol biosynthetic capacity.

### Polymorphisms of Molecular Markers

Based on the predicted *STS* gene sequences of the 12x grapevine PN40024 genome, 79 SSR primers were designed. These 79 markers were analyzed in the 95 grapevine accessions. Forty SSR markers showed higher polymorphism, and 123 alleles were identified. The PICs of the SSR loci ranged from 0.0206 to 0.6712, with an average of 0.2877 (Supplementary Table S3).

### SSR Analysis

**FIGURE 2:**
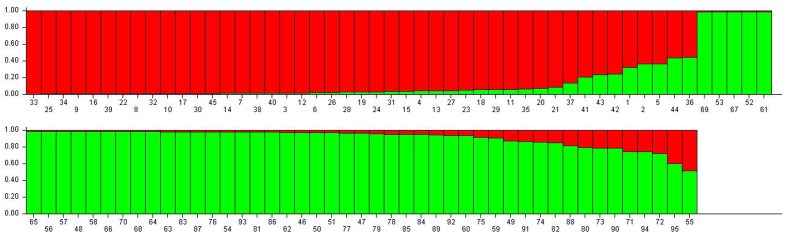
**Population structure of the 95 grapevine accessions**. The numbers represent plant material according to **Table 1**. Population one (P1, red) included 45 table and wine grapes, whereas Population 2 (P2, green) included 50 wild Chinese grapevine accessions.

**FIGURE 3:**
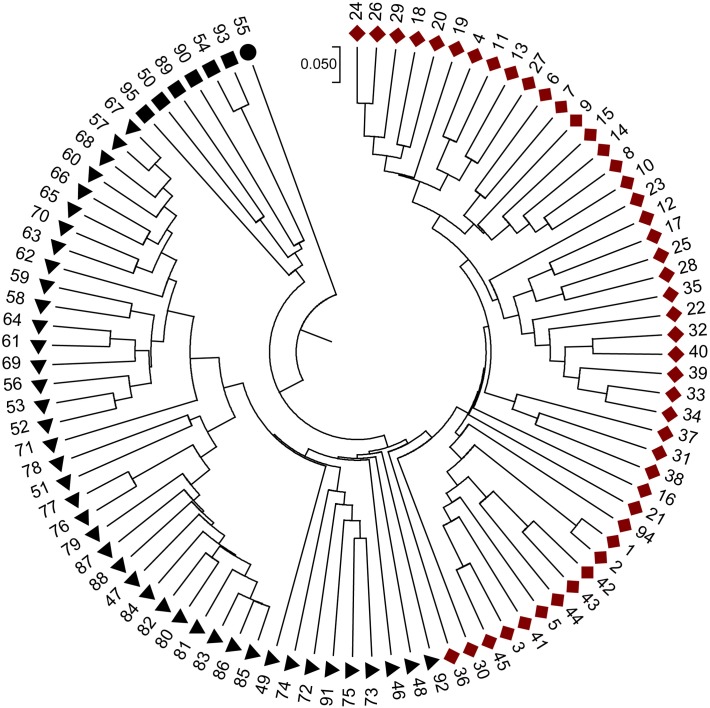
**Phylogenetic relationships of the accessions based on genetic distances calculated using SSR data and UPGMA clustering constructed using MEGA 6.0 software**. Four icons, 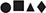, represent four sub-divisions. Red and black represent two main groups, respectively.

When the STRUCTURE software was run using all 95 grapevine accessions, the delta *k* showed a significant peak when *k* = 2; thus the grapevine accessions were divided into two populations, termed P1 and P2 (**Figure 2**). This division of the population was supported by statistical probability and could ensure the accuracy of association analysis with minimum false association. P1 included 45 grapevine cultivars, both table and wine grapes, whereas P2 included 50 accessions, all of which were wild Chinese grapevine accessions (**Figure 2**). A phylogenetic tree was constructed by UPGMA analysis based on genetic distances calculated from the SSR data of the 95 accessions (**Figure 3**). Due to sufficient variability, all selected accessions were discriminated. The accessions clustered into two main groups, with six accessions (Nos. 50, 54, 89, 90, 93, and 95) forming a third, distinct cluster (black square). All accessions formed a branch with other accessions and cultivars, except two, namely *V. davidii* accession Dongxiangjiao (No. 55, black circle), which did fall in close to another branch, and *V. yeshanensis* accession Yanshan (No. 94), which did not sort into near wild grapevines. This corresponded to the evaluated populations with STRUCTURE software, with a few exceptions. The above SSR analysis generally agreed with the geographic origins and pedigree of the grapevine accessions.

### Association Analysis between Resveratrol and SSR Marker Loci

**FIGURE 4:**
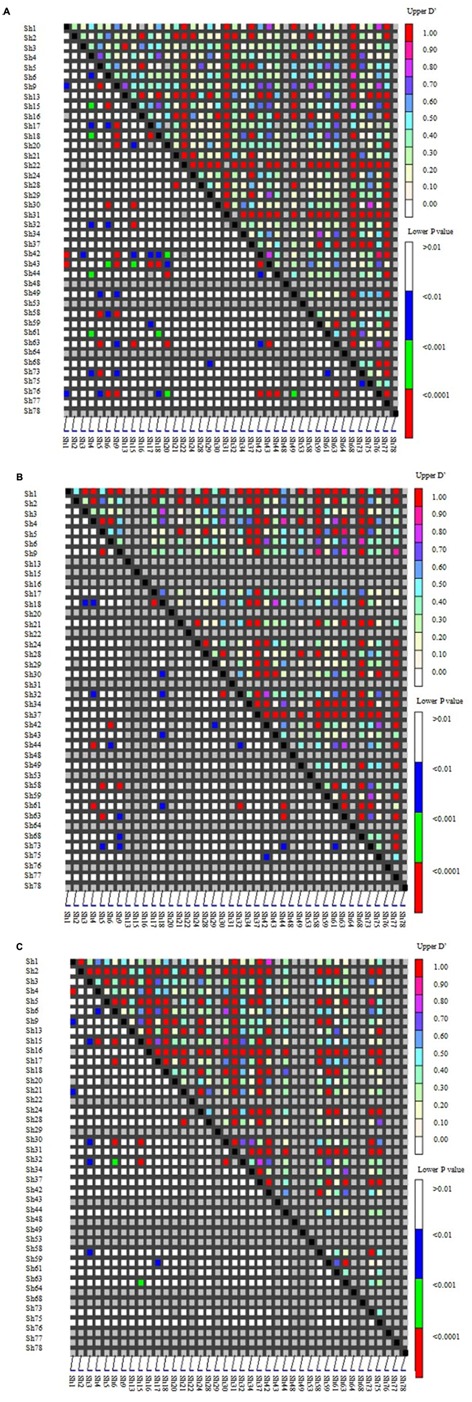
**Distribution of Linkage disequilibrium (LD) among 40 SSR loci in two groups of 95 grapevine accessions**. SSR markers are organized in linkage groups marked along the *X*- and *Y*-axis; each pixel above the diagonal indicates the D′value of the corresponding marker pair as shown in the color code at the upper right, while each pixel below the diagonal indicates the *p*-value size of the testing LD of the corresponding marker pairs as shown in the color code at the lower right. **(A)** Distribution of LD in 95 grapevine accessions. **(B)** 50 wild Chinese accessions. **(C)** 45 grapevine cultivars.

Linkage disequilibrium (LD) among genes was the basis of the association analysis. Distribution of LD among the 40 SSR loci in the two groups (according to **Figure 2**) was shown as **Figure 4A**. Loci with high LD values (D′ > 0.7; upper right corner) were Sh13, Sh16, Sh22, Sh31, Sh37, Sh68, and Sh78.The LD among the wild Chinese grapevines (**Figure 4B**) was significantly higher than those of the grapevine cultivars (**Figure 4C**, including table grapes and wine grapes). The mean frequency distribution of the D′ value (*P* < 0.001) was 0.5329 for all experimental samples (**Table 2A**), 0.6046 for the *V. vinifera* cultivars, and 0.7037 for the wild Chinese accessions (**Table 2B**). The higher D′ in the wild population indicates more variation. In addition, the number of LD loci among the grapevine cultivars was fewer than in the wild Chinese accessions (**Table 2B**).

**Table 2:** The frequency distribution of D′ value.

(A) Linkage disequilibrium (LD) for pairwise SSR loci among all 95 grapevine accessions.
**Number of LD locus pairs**	**Frequency distribution of D′ (*P* < 0.001)**					
	**0–0.2**	**0.2–0.4**	**0.4–0.6**	**0.6–0.8**	**0.8–1.0**	**Mean of D′**

68 (8.72%)	0	14	32	20	2	0.5329

**(B)** **Comparison of LD values for pairwise SSR loci between *V. vinifera* cultivars and wild Chinese grapes**.						

**Population**	**Number of LD locus pairs**	**Frequency distribution of D′ (*P* < 0.001)**					
		**0–0.2**	**0.2–0.4**	**0.4–0.6**	**0.6–0.8**	**0.8–1.0**	**Mean of D′**

*Vitis* cultivars	18 (2.44%)	0	2	6	8	2	0.6046
Wild Chinese grapes	28 (3.59%)	0	0	9	11	8	0.7037

Based on LD analysis and the current suitable population, association analysis was performed with candidate markers using Tassel 2.1 software. Eight SSR loci, namely Sh5, Sh9, Sh21, Sh28, Sh56, Sh63, Sh76, and Sh77, were significantly (*P <* 0.001) associated with resveratrol content and their explained phenotypic variation (EPV) were all higher than 10% (**Table 3**). Loci Sh5, Sh21, Sh28, Sh63, and Sh76 were associated with high resveratrol content in the leaves, whereas loci Sh5, Sh9, Sh56, and Sh77 were associated with high resveratrol in berry skins (**Table 4**). Moreover, these associations were independent of the year. Locus Sh5 was associated with high resveratrol content in both tissues in both seasons.

**Table 3:** Marker loci associated with resveratrol content and their explained phenotypic variation (significance at *P <* 0.001).

Trait	Locus	p_Marker	EPV (%)
Leaf in 2013	Sh5	0.00044317	0.1891
	Sh21	0.00034856	0.1916
	Sh28	0.00008824	0.1922
Leaf in 2014	Sh5	0.00080000	0.1429
	Sh63	0.00001241	0.2018
	Sh76	0.00000073	0.2760
Skin in 2013	Sh5	0.00000027	0.3121
	Sh9	0.00098624	0.1939
	Sh56	0.00069694	0.1187
	Sh77	0.00000003	0.2850
Skin in 2014	Sh5	0.00000028	0.3033
	Sh9	0.00050000	0.1800
	Sh56	0.00062219	0.1172
	Sh77	0.00000001	0.2930

**Table 4:** Phenotypic effects of some marker alleles at loci significantly associated with resveratrol content.

Trait	Locus	Allele size (bp)	Phenotypic effect
Leaf in 2013	Sh5	232	17.49
		236	-1.81
	Sh21	264	-4.48
		266	-4.92
	Sh28	220	-4.09
		222	-3.93
Leaf in 2014	Sh5	232	10.43
		236	-10.11
	Sh63	120	-6.17
		122	-5.82
		124	-5.89
	Sh76	113	-3.34
		115	-4.02
Skin in 2013	Sh5	232	19.28
		236	-0.19
	Sh9	239	18.05
		243	0.24
		247	2.77
		253	0.10
		256	1.30
	Sh56	125	1.45
		129	8.60
	Sh77	117	-38.45
Skin in 2014	Sh5	232	-1.50
		236	-3.12
	Sh9	239	20.06
		243	2.49
		247	4.23
		253	2.08
		256	2.99
	Sh56	125	1.28
		129	8.99
	Sh77	117	-39.90

The phenotypic effects of the different alleles of the eight loci significantly associated with resveratrol content were evaluated (**Table 4**). Allele 236 at locus Sh5 produced negative effects four times. On the other hand, allele 232 produced positive effects three times, including in leaves in the 2 years. Loci Sh9 and Sh56, through seven alleles, exhibited only positive effects in berry skins, whereas one allele of locus Sh77 created negative effects in berry skins in the 2 years. The rest of the loci showed negative effects at least once.

The eight loci significantly associated with resveratrol content were mapped to the 12x grapevine PN40024 genome. This revealed that locus Sh56 (location 16506665–16506789 on Chromosome 16) was closely linked to *VvSTS27* (16507444-16503155) and that locus Sh77 (16:366055-16:366171) was closely linked to *VvSTS17* (16372414-16366426) (**Table 5**). The other six loci were not very closed to known *STS* genes. However, future investigation of predicted genes at these loci may reveal their functions in secondary metabolism.

**Table 5:** Repeat motif and physical location of eight SSR loci significantly associated with resveratrol (*P* < 0.001) on the 12x grapevine PN40024 genome.

Primer name	Motif	No. of Repeats	PN40024 12 X location
Sh5	at	13	16323230 16323465
Sh9	tat	7	16320838 16321080
Sh21	at	26	16247793 16248056
Sh28	ga	5	16257727 16257946
Sh56	at	4	16506665 16506789
Sh63	ag	6	16630877 16631000
Sh76	tc	10	Chr16:363088 Chr16: 363201
Sh77	tc	4	Chr16: 366055 Chr16: 366171

## Discussion

Grapevine is one of the most important fruits in the world. Table grapes are a healthy snack, grape leaves are a staple in some diets, and wine grapes produce a favorite beverage. Resveratrol in both berries and leaves benefit human health, an attribute which has attracted widespread interest. Breeders aim to select and improve the content of resveratrol and other secondary metabolites, such as stilbenes, in grape. Moreover, stilbene concentrations vary depending on multiple factors, including grape cultivar, fungal infection, and climate condition (Jeandet et al., 1995; Mattivi et al., 1995; Ribeiro de Lima et al., 1999). In the present study, the resveratrol contents in 95 accessions were determinated by HPLC method in two growing seasons. *Trans*-resveratrol content ranged from 0.03 to 68.44 μg⋅g^-1^FW in berry skins and from 0.04 to 11.69 μg⋅g^-1^ FW in leaves. A previous study found that resveratrol was significantly higher (1) in berry skin of seeded cultivars than of seedless ones; (2) in berry skin and seeds in wine grapes than in table grapes; (3) and in red grapes than in green (Li et al., 2006). A recent study reported that an *STS* allele from the wild Chinese grapevine *V. pseudoreticulata* could confer accumulation of stilbenes and resistance against powdery mildew in an *Arabidopsis* heterologous system, whereas the allele from *V. vinifera* ‘Carigane’ could not be expressed (Jiao et al., 2016). Together these results demonstrate a wide range of resveratrol content in wild, table and wine grapes, which also suggests the existence of potential genetic variation for resveratrol biosynthesis. Therefore, the use of a wide collection of 95 grapevine accessions in our study lays a foundation for finding elite alleles for resveratrol production.

*STS* genes encode key enzymes in the last stage of resveratrol biosynthesis. In grapevine, the *STS* gene family contains at least 40 members, although most relevant studies thus far have focused on only one or two *STS* genes from grapevines and peanuts. Overexpression of *STS* genes can improve resistance against a fungal pathogen and other abiotic stresses and increase either resveratrol accumulation (Zhu et al., 2004; Kiselev and Aleynova, 2016), or piceid accumulation (Ruhmann et al., 2006; Liu et al., 2011; Carlos-Hilario et al., 2015). The expression of 32 *STS* genes was analyzed after exposure to UV light, and function of nine *STS* genes of them was characterized (Parage et al., 2012). Our previous findings also showed that about 40 *STS* genes had different expression patterns in different tissues and environments (Shi et al., 2014). Members of the *STS* gene family were analyzed for differences in their molecular structure and transcript accumulation (Vannozzi et al., 2012). In the present study, 40 SSR loci with high polymorphism (an average of 0.2877) were located on Chromosome 16 of the grapevine PN40024 genome, suggesting that Chromosome 16 may be more responsible for resveratrol biosynthesis than *STS* genes on other chromosomes.

Through correlation analysis, all representative samples of the population and the polymorphisms of the SSR markers link an associated locus to several allelic variants. If the corresponding allelic variation tends to phenotypic diversity, it might be selected as optimal allelic variation. In the present study, 8 SSR loci were significantly (*P* < 0.001) associated with resveratrol content, with EPV higher than 10%. Of them, four loci showed repeated associations in four environments. Locus Sh5 associated with high resveratrol content four times, with allele 232 linked three times for positive effects, including in leaves in the 2 years. But allele 236 showed negative effects four times. For resveratrol content in berry skins, loci Sh9 and Sh56, with a combined seven alleles, exhibited positive effects. Recently, many studies using molecular markers have amplified multiple bands, identified relationships, mapped markers to chromosomes, and analyzed the association between molecular markers and agronomic traits (Abdurakhmonov et al., 2008; Jahnke et al., 2011; Lorenzis et al., 2013; Liu et al., 2014; Cai et al., 2016). However, there have not been many studies on the markers of selected genes (Jin et al., 2016).

As resveratrol is directly catalyzed by STS, correlation between known *STS* alleles, our SSR markers, and resveratrol content were sought. We found eight loci with significant association to resveratrol content in a wide grapevine germplasm collection, while controlling false positives potentially deriving from population structure and multiple testing. Three SSR loci in berry skins with positive effects were mapped onto Chromosome 16. These loci were close to *VvSTS17* or *VvSTS27*. These findings can inform future use of grapevine germplasm resources in breeding for production of resveratrol and its derivatives.

## Author Contributions

XZ and JS contributed equally to this work. JF, XZ, and JS: conceived and designed the experiments. JS, YY, and YS: performed the experiments and analyzed the data. XZ, JS, and YY: contributed reagents/materials/analysis tools. JF, BT, XY, and JL: provided guidance for the entire study. JS: wrote the manuscript. All authors approved the final manuscript.

## Conflict of Interest Statement

The authors declare that the research was conducted in the absence of any commercial or financial relationships that could be construed as a potential conflict of interest.

## Footnotes

1 http://www.genoscope.cns.fr/externe/GenomeBrowser/Vitis/

2 http://archive.gramene.org/db/markers/ssrtool

3 http://www.chinadps.net/download.html

4 http://www.megasoftware.net

5 http://pritch.bsd.uchicago.edu/software/structure21.html

6 http://www.maizegenetics.net/tassel/tassel2.1_standalone.zip
