# Supplementary material for: Exploration of Elite Stilbene Synthase Alleles for Resveratrol Concentration in Wild Chinese Vitis spp. and Vitis Cultivars
Source: Front Plant Sci. 2017 Apr 7;8:487. doi: 10.3389/fpls.2017.00487 (PMC5383651; doi:10.3389/fpls.2017.00487)
Supplement: Supplementary file 1 [file Table_1.pdf]

Supplementary Table S1 Climate data in Zhengzhou (the National Grape Germplasm Resources Repository) in 2013 and 2014

Year		2013	2014
Mean temperature	June	26.2	26.3
(℃)	July	29.1	28.8
	August	30.1	25.8
	September	23.5	21.2
Relative humidity	June	53	56
(%)	July	66	61
	August	54	69
	September	55	79
Rainfall	June	20.2	27.9
(mm)	July	45.1	50.3
	August	63.9	67.7
	September	9.8	228.0

Notes: The data were from the National Grape Germplasm Resources Repository of Zhengzhou Fruit Research Institute,

Supplementary Table S2 The variation of resveratrol content in four environments. ND means not detected using HPLC method.

A. The variation of resveratrol content of 95 accessions.

Variation	In skins		In leaves	
	Number	Percent	Number	Percent
≥100%	5	5.26%	14	14.74%
＜100% and≥50%	22	23.16%	27	28.42%
＜50%and≥20%	15	15.79%	12	12.63%
＜20%and≥10%	13	13.68%	18	18.95%
＜10%	37	38.95%	15	15.79%
ND	3	3.16%	9	9.47%

Notes: Variation =│Resveratrol amount in 2014－Resveratrol amount in 2013| /Resveratrol amount in 2013*100%.

Percent = the Number/95*100%.

B. The variation of resveratrol content of 45 cultivars.

Variation	In skins		In leaves	
	Number	Percent	Number	Percent
≥100%	4	8.89%	4	8.89%
＜100%and≥50%	19	42.22%	26	57.78%
＜50%and≥20%	5	11.11%	6	13.33%
＜20%and≥10%	5	11.11%	5	11.11%
＜10%	9	20.00%	2	4.44%
ND	3	6.67%	2	4.44%

Notes: Variation =│Resveratrol amount in 2014－Resveratrol amount in 2013| /Resveratrol amount in 2013*100%.

Percent = the Number/45*100%.

C. The variation of resveratrol content of 50 wild accessions.

Variation	In skins		In leaves	
	Number	Percent	Number	Percent
≥100%	1	2.00%	10	20.00%
＜100%and≥50%	3	6.00%	1	2.00%
＜50%and≥20%	10	20.00%	6	12.00%
＜20%and≥10%	8	16.00%	13	26.00%
＜10%	28	56.00%	13	26.00%
ND	0	0.00%	7	14.00%

Notes: Variation =│Resveratrol amount in 2014－Resveratrol amount in 2013| /Resveratrol amount in 2013*100%.

Percent = the Number/50*100%.

Supplementary Table S3 The sequence and genetic characterization of 40 SSR markers from the grapevine PN40024 genotype.

Abbreviations: the number of alleles (Na), effective number of alleles (Ne), observed heterozygosity (Ho), and expected heterozygosity (He), and polymorphism information content (PIC).

Primer Name	Forward primer (5'-3')	Reverse primer (5'-3')	Excepted product (bp)	Na	Ne	Ho	He	PIC
Sh1	TATAAGCAATGGGTAA	TATTTTGATAGGGATG	282	4.0000	1.5842	0.0108	0.3708	0.3213
Sh2	ACTTATCCTTTAGGGTTA	CTAAATACAATTGGAAA	275	3.0000	1.1483	0.0947	0.1298	0.1239
Sh3	AACCCTATACATACCCTT	CTAACTTAGATATTCCCAGA	239	4.0000	1.6288	0.0737	0.3881	0.3581
Sh4	GGCTACATTAAGTAACCT	GGATATGGAATAACAAAG	245	2.0000	1.7115	0.1474	0.4179	0.3293
Sh5	GATCTAATTTACGGTCAC	TTAGAAGAATATGGTGGA	236	4.0000	1.1896	0.0638	0.1603	0.1534
Sh6	CAAAGTAGAAGAGTGCAACA	CCAACTTCCATAAACCTC'	219	6.0000	1.9507	0.2211	0.4899	0.4648
Sh9	TTCAAAGTTTTCAAGCATC	TTTCCCTGGGCCTTCTAT	243	5.0000	3.1577	0.3407	0.6871	0.6447
Sh13	AATTGAGATAACGCCTAG	TCTTCCCTGATAATAGTG	213	3.0000	1.1005	0.0947	0.0918	0.0894
Sh15	AGGTGTTGCCTGATTGTG	TTTTAGGTCGCTTAGATG	276	3.0000	1.2858	0.2500	0.2235	0.2038
Sh16	TTTGGTGGGCTCAAGTTA	ATTTGAACAAAGTCATAGGTTGTCT	228	3.0000	1.8921	0.7097	0.4740	0.3701
Sh17	ATTTGCCCATGTTAGTAA	ATCCTCATTGATTTTAGC	172	4.0000	2.2220	0.0323	0.5529	0.4750
Sh18	AATACTCAGTTTTAAG	TACGAAATGAGTCTAA	126	2.0000	1.7317	0.0319	0.4248	0.3330
Sh20	TAGAAAACGACAAAAC	GTTCACATAGGTCCAC	205	4.0000	3.1564	0.3333	0.6876	0.6257
Sh21	ACTAACTTAGAAGGTGCT	TAAACTATGTGAGGAGAA	264	4.0000	1.1990	0.0737	0.1669	0.1578
Sh22	CATGGACTGTGATTTGTT	TAGCATCTCAAGCTCAAC	240	2.0000	1.2800	0.2167	0.2206	0.1948
Sh24	CATACATCACATAACCCTT	TTGAAGCATTGGATTACT	247	2.0000	1.1824	0.1053	0.1551	0.1423
Sh28	TCTCAATAAACCAACTCT	ACGTGGCTCTTATAATAC	220	3.0000	1.3024	0.2632	0.2334	0.2116
Sh29	TTTAGACAACCCATTAGA	TTAACGCAAGCTAAGTAT	212	2.0000	1.5654	0.3226	0.3632	0.2960
Sh30	CATACCTGGATAGCGAACA	GACCCTTGTTGCATGTTT	263	2.0000	1.3489	0.3053	0.2600	0.2252
Sh31	ATATTACAATACCAAAGCC	TAGGAGAAGTTCCCTAAA	186	3.0000	1.1350	0.1263	0.1196	0.1131
Sh32	TGAGAAGGTCCAGAACTA	GACTTCTTGAAAATGGTT	222	5.0000	3.5732	0.1183	0.7240	0.6712
Sh34	GGAGGGTCTACATCGTGA	AACCTTACACTTGGCACA	253	3.0000	2.0849	0.5532	0.5232	0.4514
Sh37	CAATTTTAAGAGCTACAC	AAATGAGTCTAATTCCAA	153	2.0000	1.8705	0.6737	0.4678	0.3571
Sh42	ATGCAACAGATAAACCTC	CTTCGAAAACATAAAACA	226	3.0000	2.0703	0.0638	0.5197	0.4036
Sh43	TTTAGAAATGTGGCATAGAG	CCTTGAGGATAGGGAGAA	221	2.0000	1.9919	0.2340	0.5006	0.3740
Sh44	TGTAAGATCCATCCTGAG	GTGCTGATAATGTATTGTAA	248	5.0000	3.1169	0.5000	0.6831	0.6407
Sh48	AAATGGGTCCCAATGAAG	CAAGATGGGTGATAGTAAAT	211	2.0000	1.0213	0.0211	0.0209	0.0206
Sh49	AAAGTTATGCAAGCTCTG	GTCTTAATCCAACTCACG	177	3.0000	1.2516	0.2211	0.2021	0.1887
Sh53	CCCACCTGAATTCCACTCATTT	ACCTCCTCAAAGACCTCAAGTT	158	3.0000	1.1835	0.1684	0.1559	0.1446
Sh56	AGACACCTACTCAATCCATTGC	ATCATTTCCCATACAGGTCCTT	125	3.0000	1.1591	0.1474	0.1380	0.1292
Sh59	CCTTCCATTATCCAACATTCTC	GAGGGTAGGAGGGTCTATTTC	128	3.0000	1.2985	0.2632	0.2311	0.2058
Sh61	GGAACCTGGAGTCTCATCCTT	GGCTTGACCATTGAAACTGTTG	254	2.0000	1.4297	0.3684	0.3021	0.2554
Sh63	CCAGACAAGAGAATGAGCAGAG	GGTATCCCGCAACAATGTAGAA	124	4.0000	1.7948	0.2421	0.4452	0.4034
Sh64	CCGACGGTGCTACGATAGA	GTGATCTTGGACGCCTGAATT	217	2.0000	1.0651	0.0632	0.0615	0.0593
Sh68	ACCATCGTTGTTCGTGAATAAG	AGCAGAATTGGAGAATCAGTGT	163	2.0000	1.0430	0.0000	0.0414	0.0405
Sh73	TGAGGTCACTACACCTTCTAAC	GGACCAGGCTTAACCATTGAA	233	4.0000	2.5024	0.9158	0.6036	0.5195
Sh75	CTTCCTCATCCTCATCCATT	GAGTTCTCAGGTGCCAAA	271	3.0000	1.5146	0.0842	0.3416	0.3137
Sh76	CTCTTCCTCCTCCACTCT	GCCCACCTTGTTTGAAAT	113	3.0000	2.1421	0.0952	0.5364	0.4242

Sh77	TTGAACCAGATGAGAAACCA	CGACCGATTGAGGCTTAA	117	2.0000	1.0321	0.0105	0.0312	0.0306
Sh78	TGGATGATGTATGTCTCACA	GGTAACACAATGTTTGCTCT	159	2.0000	1.0430	0.0421	0.0414	0.0405
			**Total**	123			Mean	0.2877
